# One Hundred Years of Migration Discourse in *The Times*: A Discourse-Historical Word Vector Space Approach to the Construction of Meaning

**DOI:** 10.3389/frai.2020.00064

**Published:** 2020-09-10

**Authors:** Lorella Viola, Jaap Verheul

**Affiliations:** ^1^Luxembourg Centre for Contemporary and Digital History (C^2^DH), University of Luxembourg, Esch-sur-Alzette, Luxembourg; ^2^Department of History and Art History, Utrecht University, Utrecht, Netherlands

**Keywords:** word-vector space, migration discourse, historical newspapers, critical discourse analysis, diachronic conceptual change, language & media, migration history

## Abstract

This study proposes an experimental method to trace the historical evolution of media discourse as a means to investigate the construction of collective meaning. Based on distributional semantics theory (Harris, 1954; Firth, 1957) and critical discourse theory (Wodak and Fairclough, 1997), it explores the value of merging two techniques widely employed to investigate language and meaning in two separate fields: neural word embeddings (computational linguistics) and the discourse-historical approach (DHA; Reisigl and Wodak, 2001) (applied linguistics). As a use case, we investigate the historical changes in the semantic space of public discourse of migration in the United Kingdom, and we use the *Times Digital Archive* (TDA) from 1900 to 2000 as dataset. For the computational part, we use the publicly available TDA word2vec models^1^ (Kenter et al., 2015; Martinez-Ortiz et al., 2016); these models have been trained according to sliding time windows with the specific intention to map conceptual change. We then use DHA to triangulate the results generated by the word vector models with social and historical data to identify plausible explanations for the changes in the public debate. By bringing the focus of the analysis to the level of discourse, with this method, we aim to go beyond mapping different senses expressed by single words and to add the currently missing sociohistorical and sociolinguistic depth to the computational results. The study rests on the foundation that social changes will be reflected in changes in public discourse (Couldry, 2008). Although correlation does not prove direct causation, we argue that historical events, language, and meaning should be considered as a mutually reinforcing cycle in which the language used to describe events shapes explicit meanings, which in turn trigger other events, which again will be reflected in the public discourse.

## Introduction

The emergence of unprecedented masses of digital data has brought an upsurge in Natural Language Processing (NLP) studies concerned with language and meaning. These studies today are mostly based on distributional semantics theory (Harris, 1954; Firth, 1957) and typically use techniques such as neural word embeddings to map different senses expressed by single words. However, some computational linguists have observed that, with the exception of few recent initiatives that go beyond single words^2^, most current methods have failed to adopt more holistic approaches. A recent survey of studies on lexical semantic change detection (i.e., Tahmasebi et al., 2018), for instance, has indicated that the “issue of interdependence between semantic changes of different words” remains largely unexplored (Tahmasebi et al., 2018, p. 42). This would be due to that fact that works on lexical semantic change based on neural word embeddings have almost exclusively investigated single words. According to these studies, meaning change should on the contrary be understood as belonging to “an intricate net of word-to-word interrelation” as the focus on single words does not allow for a comprehensive view of how a given word changes meaning. This may suggest that, rather than looking at word senses separately, whole concepts or topics should be the focus of inquiry so that meaning changes are studied in the context of other words that express (or used to express) the same or related concepts.

Another exciting challenge that the field of NLP on language and meaning still presents concerns the scope of the inquiry. Kutuzov et al. (2018), for instance, have pointed out how the investigation continues to be concerned more with proving that a change has happened, rather than with identifying potential explanations for it. These authors, for instance, noted that “a more detailed analysis of the nature of the shift is needed,” and similar to Tahmasebi et al. (2018), this could be accomplished through the identification of “groups of words that shift together in correlated ways,” or with “identifying the source of a shift” (Tahmasebi et al., 2018, p. 1393), for instance, by studying linguistic or extralinguistic causes.

The availability of unprecedented masses of digital data has also brought the issue of interdisciplinarity between the humanities and the sciences at the center of the academic debate. It is certainly true that over the past few years, we have witnessed a growing number of studies that have combined approaches and methods from both fields. In disciplines such as linguistics and history, for example, the digital turn has called for a reconceptualization of the practice, almost forcing scholars to adopt advanced quantitative methods in their research, whereas in the humanities at large, it has led to the emergence of completely new fields such as digital humanities. Conversely, in computational linguistics, scholars have increasingly integrated linguistic theories and social data into their models, allowing for an increase in performance as well as significant advances, even in neighbor fields such as machine learning. Despite the major achievements, however, scholars across both sides have expressed a need for more integrated methods to combine perspectives, as well as accelerate and expand knowledge, as they feel that currently expertise remains essentially disconnected (e.g., Jockers, 2013; Snow, 2013; Tahmasebi et al., 2018). This struggle to actualize interdisciplinarity, particularly in the humanities, is perhaps best reflected in the difficulty to combine close reading with distant reading (Viola and Verheul, 2019). While computer science delivers solutions to automate or semiautomate analytical processes, close-reading approaches continue to be largely preferred in linguistics and the humanities. At the same time, scholars, particularly those working in digital humanities, have attempted to blend both approaches upon the conviction that “quantitative methods are most effective when used alongside the close textual reading” (Gooding et al., 2012, 2013). Jockers (2013), for instance, suggests what he calls a “macroanalysis” approach, whereas Graham et al. (2016) propose a workbench of different tools, called the “historians' macroscope.” Similarly, Lee (2019) uses a range of distant reading techniques to automate a large part of the data preprocessing destined to critical discourse analysis (CDA) investigations, and finally, Viola and Verheul (2019) argue for a merged method, “discourse-driven topic modeling,” effective at uncovering and making sense of historical patterns in large quantities of textual data. What these scholars have essentially tried to achieve is a mutual compensation for the limitations of both distant and close reading approaches: the need for an analytical contextualization of quantitative findings of the former and the impossibility of critically reading everything of the latter (Viola and Verheul, 2019).

This study aims to address such current challenges by exploring the value of merging two different techniques widely employed in two separate fields: neural word embeddings, a quantitative, distant reading method used in computational linguistics to investigate meaning change; and the discourse-historical approach (DHA; Reisigl and Wodak, 2001), a qualitative, close reading methodology used in applied linguistics to investigate the relationship between language and discourse. With this experimental method, our goal is to widen the focus of the analysis from identifying that a word has changed its meaning over time to exploring plausible extralinguistic factors that may reveal the mechanisms involved in the construction of collective meaning. As a use case, we investigate the historical changes in the semantic space of public discourse of migration in the United Kingdom, and we use the *Times Digital Archive* (TDA) from 1900 to 2000 as dataset. For the computational part, we use the publicly available TDA word2vec models (Kenter et al., 2015; Martinez-Ortiz et al., 2016); these models have been trained according to sliding time windows (cfr. *Methodology and Dataset*) with the specific intention to map conceptual change. We then use DHA to triangulate the results generated by the word vector models with social and historical data in order to provide plausible clarifications of the processes underpinning the construction of meaning itself. By merging these two complementary techniques, we hope to bring together different research modalities that could provide scholars with a research tool informed by critical, methodological, and empirical approaches (Berry and Fagerjord, 2017, p. 104).

The proposed method should not be seen as necessarily “better” than others; instead, our intention is to propose a more holistic approach that goes beyond simply identifying meaning changes and that could provide avenues for understanding the mechanisms underlying the construction of meaning in relation to language and public discourse. By integrating the quantitative findings with a discourse-historical interpretation, the method intends to add the currently missing sociohistorical and sociolinguistic depth to the computational results. This combination allows us to see how public discourse around the topic of migration has changed over the course of one century and how such changes may reflect the underlying sociohistorical events. We argue that this kaleidoscopic approach may reveal more than just the occurrence of a change in meaning: the method may help us to discover why such change has occurred at a given time and, more widely, how meaning is collectively constructed.

## Language and The Construction of Meaning

The study of the relationship between language and meaning has long been the interest of many disciplines, including philosophy, psychology, anthropology, history, literature, linguistics, and, more recently, computational linguistics. As a result, authors have proposed a wide range of terminologies, classifications, and definitions. Although these publications have been influential in deepening our understanding of the complexities of word meaning, they also produced a multiplicity of labels and taxonomies that sometimes caused disagreement. For instance, there is no consensus on the outstanding question whether we should theoretically distinguish between *meaning* and *concept* or whether we should use these terms interchangeably. In fields such as conceptual history, for example, *concepts* seem to have been considered simply as words (Tahmasebi et al., 2018, p. 3). This would be reflected in the argument that the change in the vocabulary of specific terms indicates a change in the way the respective societal groups use such terms (e.g., Brunner et al., 1972; Koselleck, 1992, 2004); hence, in this field, definitions of *concept* are typically fuzzy and contested (Margolis and Laurence, 2005; Tahmasebi et al., 2018). This nevertheless important work conducted by conceptual historians on conceptual change (e.g., Skinner, 1969, 1978, 2012; De Bolla, 2013; Gavin, 2015; Recchia et al., 2017; De Bolla et al., 2019) has primarily searched for evidence of variation (i.e., vocabulary change), which is looked at through the lens of historical events or long-term changes in social stratification of society (Koselleck, 2004). Therefore, fundamental alterations in the meaning of keywords are interpreted as the reflection of conceptual changes (Pocock, 2016). We aim to expand on such studies by widening the scope of the investigation and focusing on what changes in society and language tell us about how meaning itself is constructed, which has often fallen beyond the scope of historical inquiry. We argue that this approach may build an interdisciplinary bridge between linguistics and conceptual history in studying the relation between changes in language and those in society.

Historical linguists, too, have identified culture as a crucial factor in language change; at the same time, however, they also point at even more fundamental mechanisms that trigger language change, such as for example, language contact. Linguists believe that before looking at potential changes in the meaning of words and what such changes might reveal, it is first essential to ask what meaning is in relation to language and society. In this sense, the most substantial theoretical and empirical contribution is offered by linguistics and, more recently, by modern empirical linguistics (i.e., corpus linguistics and computational linguistics). For this reason, we will here only refer to work within these fields that has attempted to address these questions.

### Linguistic Approaches to the Study of Language and Meaning

Before the advent of powerful computers and the availability of large historical linguistic datasets, language was mostly studied through invented examples, using a speculative, intuitive approach. With the exception of few sporadic pioneering initiatives, for instance, in dialectology (i.e., Wenker, 1878) or in the first wave of variation studies (e.g., Labov, 1966; Trudgill, 1974; Macaulay, 1977), linguists primarily used introspective language competence and perception to formulate theories of what was possible and not possible in a language. Typically, they would formulate explanatory theories to describe certain phenomena and then invent examples that would confirm those theories. Because gaining access to real language data was costly and very time-consuming, their knowledge of the language as native speakers was the preferred “data” they would use. Consequently, these imagined examples of what was *possible* in a language were generalized to the language as a whole and considered as *real*. As most of the time they did not have the possibility to test invented examples on real-use data other than their internalized knowledge of the language, it was generally accepted that *possible* usage meant *real* usage.

Corpus linguistics has changed this tradition. Thanks to the analysis of billions of sentences of real-use language, we now know that languages are not deterministic systems, but rather they should be thought to be “probabilistic, analogical, preferential systems” (Hanks, 2013, p. 310). This ground-breaking discovery called for a review of many previous linguistic theoretical formulations and earlier established assumptions. New advances in computer science merged with huge quantities of digital material, including historical datasets, have allowed modern empirical linguists to study how people use words to communicate much more rapidly and efficiently than ever before. Today, the unprecedented amounts of naturally occurring language data have originated linguistic subfields such as corpus-based historical pragmatics and semantics and computational sociolinguistics. These disciplinary developments not only have yielded a deeper understanding of what meaning is, but also pointed at novel ways to study changes of how word meaning is constructed over time.

If certain twentieth century linguistics hypotheses have been challenged by real-use data, others have been later tested and confirmed. One such hypothesis is Firth's (1957, p. 11) famous intuition that “You shall know a word by the company it keeps.” This intuition provided the foundation for his work on collocational meaning, which acknowledges the relevance of collocation in determining meaning. Collocational meaning has substantially contributed to the field of distributional semantics, the field of study concerned with measuring and categorizing how words are used based on patterns of usage. The core idea of distributional semantics is that meanings do not exist in isolation, but rather that words that are used and occur in the same contexts tend to purport similar meanings (Harris, 1954, p. 156). The distributional hypothesis is still central to most lines of inquiry of NLP techniques and has been applied to computational word vector models, including, for instance, the word2vec algorithm of Google. What is perhaps even more relevant to work on semantic change is the firm rejection of the belief that words can have a one-to-one relationship with meaning. As Harris argued (emphasis added), “We cannot say that each morpheme or word has a single or central meaning, or even that it has a *continuous* or coherent range of meanings” (Harris, 1954, p. 152). According to this claim, then, words in isolation do not possess meaning. We can only entail meaning from context; therefore, we can only detect changes in a word's meaning by analyzing patterns of changes in the word's context.

Another linguistic field that examines the relationship between language and meaning is cognitive linguistics. This field sees language as a mental phenomenon. Accordingly, it studies language as a window on the conceptual structure of the mind and considers how the evolution of language reveals changes in the common mindset over time. One of the core principles of cognitive linguistics is that meaning involves conceptualization, i.e., *construal*. Therefore, the way language is used informs us about the construction of meaning. As Croft (2009, p. 397) puts it: “The framing of an experience through the choice of a lexical item is a matter of construal.” However, Croft himself has criticized traditional cognitive linguistics for considering language exclusively as a constellation of mental structures and processes. He argues that a comprehensive approach to language must take the fundamental function of language into account: communication. In other words, the interactive and the social dimensions of language, he claims, must be integrated in cognitive linguistics approaches to generate “a more general social–interactional model of language” (Croft, 2009). To do so, he argues, theories of pragmatics and sociolinguistics must be incorporated into cognitive linguistics.

This understanding of meaning in terms of its “discourse function” is particularly relevant to the line of research presented here. Integrating the dimension of discourse within cognitive linguistics means bringing a sociointeractional perspective to the construal of meaning, which serves “the purpose of communication” (Croft, 2009, p. 410). In agreement with Croft, our study starts from the conviction that the communicative property is essential to language and that discourse is indeed a crucial component of understanding the correlation between language, meaning, and society. Collective discourse represents the common, shared knowledge without which no communication would ever be possible. Focusing on either of the three aspects without considering their wider discourse embedding would be too restrictive and would yield only partial insights.

The approaches and theoretical frameworks discussed here, despite their differences in perspectives and goals, all agree that in the same way that meanings do not exist in isolation, discourses are not isolated entities, and that the interactive, pragmatic function of language must be considered. In our view, however, it is CDA that provides the best suited principles, theories, and methods to study language, meaning, and communication at the level of discourse. This is motivated by at least three arguments. First, critical discourse scholars define discourse as a form of social practice (Wodak and Fairclough, 1997, p. 258). Understanding discourse as a social phenomenon rather than a purely linguistic one (or mental one) entails that meaning is continually negotiated through interaction. Unlike semanticists who are concerned with the conventional meaning of words and sentences, critical discourse scholars are interested in understanding meaning as it is constructed during communication and for the purpose of communication. For critical discourse scholars, the goal is not to categorize conventional meaning, but rather to understand meaning as “socially constructed” through sign systems such as language. Discourse is in this way seen as “historically and culturally situated” rather than “eternal, absolute, and essential” (Locke, 2004, p. 11) and in a dialectical relationship with society: a discursive event shapes and is shaped by the situation, institution, and social structure that frames it (Wodak and Fairclough, 1997, p. 258).

Second, unlike conceptual historians, CDA discards a deterministic relation between discourses and society. As discourses are produced for specific purposes, their analysis entails a theorization and description not only of the social processes and structures that led to its production but also of the social mechanisms underpinning the way in which individuals or groups as social historical subjects create meanings through discourses. Therefore, because discourse is situated in time and space, CDA is particularly effective at uncovering the discursive nature of social and cultural changes (Wodak and Meyer, 2001, p. 7). This is especially true when analyzing public and media discourses, which consequently are the perfect avenues for CDA inquiry. In this respect, Wodak and Meyer (2001) notice how newsmakers often present themselves as neutral carriers of news who, supposedly through unbiased language, merely show issues of societal relevance to the public. On the contrary, CDA of media discourse has repeatedly highlighted the fundamental role of mass media in shaping meaning and discourse (*cfr. The Role of Media in the Construction of Migration Discourse*). Thus, as media are active coproducers of discourses, discourses produced via media similarly determine reality (Jäger, 2001, p. 36). According to this view (emphasis added), “discourse analysis is not (only) about interpretations of something that already exists […] but about the analysis of the *production of reality* which is performed by discourse—conveyed by active people” (Jäger, 2001).

Third, CDA provides useful methods such as the DHA that applies a sociopragmatic, historical perspective to the theory of CDA allowing to assess the historical context in which topics are formulated and discussed. In this way, the construction of meaning through language use is studied in its full sociohistorical context and as a reflection of cultural values and political ideologies. In this sense, DHA provides a triangulation of linguistic, social, and historical data that cognitive linguistics cannot offer and that is currently missing in computational semantics, unless a social cognition level is integrated into the model or a cultural one (see, for instance, Hamilton et al., 2016). In *Methodology and Dataset*, we will describe DHA in more detail.

### Computational Approaches to the Study of Language and Meaning

As it has already been said, the availability of large textual corpora and advances in computational semantics have prompted a wave of publications aimed at mapping changes in lexical semantics using distributional methods, particularly prediction-based word embedding models. This section reviews only a handful of the most recent and relevant studies, and it is by no means meant to be exhaustive^3^.

Research on detecting semantic shifts of words typically divides large historical textual corpora in time periods or “word epochs” (Mihalcea and Nastase, 2012; Popescu and Strapparava, 2014) and identifies change in the context of the word, believed to have undergone a shift by measuring co-occurring words. The Google Books Ngrams corpus proved good correlation with human judgment for detecting differences in word usage and meaning over time (Gulordava and Baroni, 2011; Kim et al., 2014; Mitra et al., 2015). In terms of sociocultural semantic shifts, a number of studies have shown that smaller time spans are more suitable, whereas longer spans should be used to study more structural, linguistic shifts. For instance, Kim et al. (2014) and Liao and Cheng (2016) used a 1 year time span dataset, whereas Kulkarni et al. (2014) applied a granularity of 1 month. These works showed the value of computational methods to trace semantic shifts with time spans of less than a decade with a particular focus on cultural drift.

Distributional word representations attempt to capture more subtle changes that may not be identified by mere word frequencies. With this technique, meaning is represented with sparse or dense (embedding) vectors, produced from continuous lexical representations of word co-occurrence counts. A number of recent publications have shown that distributional word representations (Turney and Pantel, 2010; Baroni et al., 2014) provide an efficient way to perform these tasks. Although these models still use word frequencies as data source, the information is condensed into continuous lexical representations, a technique that proved to outperform the frequency-based methods in detecting semantic shifts (Kulkarni et al., 2014).

To compare word vectors across different models Kim et al. (2014) propose the *incrementally updated diachronic embedding models*, which allow to calculate cosine similarities directly between the same word in different time period models. The technique trains a model on the diachronically first-time slice and then it updates it with the data from the successive time periods, and it saves its state each time. The intuition behind this approach is that all these models are inherently related to each other and therefore comparable.

## The Role of Media In The Construction of Migration Discourse

There is urgent consideration for understanding how the migration debate unfolds in the media. Indeed, research on the topic has been conducted in a large variety of fields (e.g., media studies, discourse studies, political communication studies, to name but a few), about myriad groups, across multiple public discourse scenarios, and over the most disparate time periods (Migration Observatory at the University of Oxford, 2016). These studies have consistently demonstrated that media play a crucial role in framing, indicating that public opinion about migration is largely informed by mass media^4^. For instance, by discussing migrants in a negative way as delinquents or criminals, media may trigger a “cultivation effect,” which slowly changes readers' perception of reality (Arendt, 2010; Balabanova and Balch, 2010; Balch and Balabanova, 2016).

Media also play an agenda-setting role, for instance, in discussing migration in the context of welfare, economy, or security (Buchanan et al., 2003; Moses, 2006, p. 137–43; Eberl et al., 2018). Multiple studies, for instance, have demonstrated how American and European media have framed migrants in a negative way, either by emphasizing the dichotomy of “us” vs. “them” or by creating an urgency of crisis (Cottle, 2000; Cisneros, 2008; Arcimaviciene and Baglama, 2018; Eberl et al., 2018; Viola and Musolff, 2019). Similarly, with reference to the UK migration debate, it has been argued that “the media are active agents in developing immigration policy” (Threadgold, 2009). Research on the more recent phase of the migration debate in the United Kingdom indicates that public perception does not match the quantitative, economic, fiscal, and cultural realities of migration (Duffy and Frere-Smith, 2014); although the global migration rates have not changed dramatically over the past half a century, on the whole in many Western nations, “the political salience of migration has strongly increased” (Lucassen et al., 2010, p. 1–4). Migration advocacy organizations and nongovernmental organizations argue that the public discussion—rather than the actual facts—plays a crucial role in creating political positions and in informing policy priorities and government choices (Sharry, 2000; Spencer, 2011; Katwala and Somerville, 2016), thus underlining the urgency of understanding how collective meaning is constructed around the migration debate.

Although UK media have generally discussed migration in negative and even dehumanizing terms (Musolff, 2015), research has also shown considerable fluctuation over time in the specific connotations, as narratives and counternarratives compete with each other in the public arena (Duffy and Frere-Smith, 2014; Burscher et al., 2015; Blinder and Allen, 2016). Race, for instance, has been discussed as a dominant context in the 1970s (Hartmann and Husband, 1974; Messer et al., 2012; Hall et al., 2013), whereas security issues started to emerge in the beginning of the twenty first century (Abbas, 2019). The variety of different voices in a wide range of media has created a complexity that is not easily resolved by close reading. Because manual content analysis is time-consuming, most of these studies have focused on a time span of a few years or a decade at most. Overall, compilations of a long-term perspective on the migration debate in UK media is still missing. For this reason, it has been argued that the field of migration studies “must move beyond thick description, single case studies, and quantification to address a set of more focused themes and questions.” (Baker et al., 2008; Gabrielatos and Baker, 2008). In a recent study (Gabrielatos and Baker, 2008), corpus analysis, i.e., collocation and word frequency, has been applied to the discursive constructions of refugees and asylum seekers in a 140-million-word corpus of UK press articles spanning one decade (1996–2015). Their analysis indeed confirmed the “media confusion and conflation of definitions” of key terms.

These works underline the promise of identifying *patterns* in the discourse over longer periods of time, currently hidden in large amounts of digital data. Our study adds to this line of inquiry and aims to achieve such goal. By combining computational, linguistic, and historical approaches, the intention is to uncover the way meaning is constructed around the urgent theme of migration in public discourse. This may also add a more quantitative perspective to migration studies, in which big quantities of data are increasingly playing a fundamental role (Pisarevskaya et al., 2019).

## Methodology and Dataset

This study proposes an experimental method that merges two techniques to trace and understand the historical evolution of media discourse. It uses word vector models trained on the TDA with a 10-year sliding window (Kenter et al., 2015; Martinez-Ortiz et al., 2016) to identify changes in the contexts in which the words *migration, immigration*, and *emigration* were used over time. We then use DHA to triangulate the word2vec results with social and historical data in order to provide plausible clarifications of the processes underpinning such changes. With this experimental method, our goal is to widen the focus of the analysis from identifying that a word has changed its meaning over time to exploring plausible extralinguistic factors that may help us understand the elements at play in the construction of collective meaning.

The word2vec models were trained on a 10 year slice with a 2 year sliding window (e.g., one model from 1900 to 1910 and sliding windows of 1900–1902, 1901–1903, 1902–1904, and so on). The word embeddings were generated with CBOW models, with 100 dimensions, a window size of 5, and minimum word count of 5, and used five negative samples. Starting from a *seed term*, the obtained terms result from the semantic network of each semantic model constructed from the documents in each time window (Kenter et al., 2015). For example, if the selected time window is 1900–1910, the outputted terms will be aggregated from the semantic models of 1900–1902, 1901–1903, 1902–1904, and so on. The weight is calculated by a Gaussian distribution where the mean of the distribution is the center of the period with a standard deviation of 1:0. The intuition behind this is that the central years in a period are the most likely to semantically reflect that period due to echoes from the preceding years and anticipations of the next years.

We compute similarities for three seed terms, *migration, immigration*, and *emigration*, in different time periods. Because the models overlap, most of the semantic relations between words remain stable, allowing us to detect granular changes over the years. As it has already been argued in the literature, this method offers a way to understand gradually changing words that are used to articulate the same topic, concept, or idea (Hamilton et al., 2016), which, in turn, allows us to trace historical changes in the construction of meaning over longer periods of time.

We use DHA to analyze the obtained similarities. The DHA applies a historical dimension to the theory of CDA (van Dijk, 1997) as it intends context as essentially historical. The historical orientation permits the reconstruction of how texts and discourses are linked intertextually and interdiscursively over time. In other words, the method considers how texts and discourses are linked to each other, both in the past and in the present, as well as to extralinguistic social/sociological variables in an effort to diachronically reconstruct and explain discursive change (Reisigl and Wodak, 2009, p. 95, 120). In practice, this is done by triangulating linguistic, social, and historical data with the aim of understanding language use as a reflection of its cultural values and political ideologies. Resulting in a quasi-kaleidoscopic investigation, this principle of triangulation arguably minimizes the risk of biases, an aspect of CDA often criticized in literature as being a method highly dependent on the researcher's interpretation. Furthermore, we argue for two additional advantages of employing the proposed integrated method: first, being supported by enormous quantities of language data such as the TDA, the combination of DHA with word vector models further contributes to reduce the risk of biases, and second, it overcomes the limitation of looking at individual documents, typical of CDA studies. Although at this stage no CDA is performed on textual excerpts, DHA is still very useful in the task of interpreting the results and explaining larger patterns.

As for the dataset, the TDA archive contains every page of every issue of the newspaper from 1785 to 2013 for a total of more than 1.6 million pages from 70,000 issues, subdivided or zoned into 11.8 million articles, cataloged by category, including advertising, editorial and commentary, news, business, news, people, and photojournalism. The subset that we used (i.e., 1900–2000) contains 5,709,334,307 tokens (i.e., all the words) and 359,351,482 words (i.e., word types).

## Analysis

**Figure 1:**
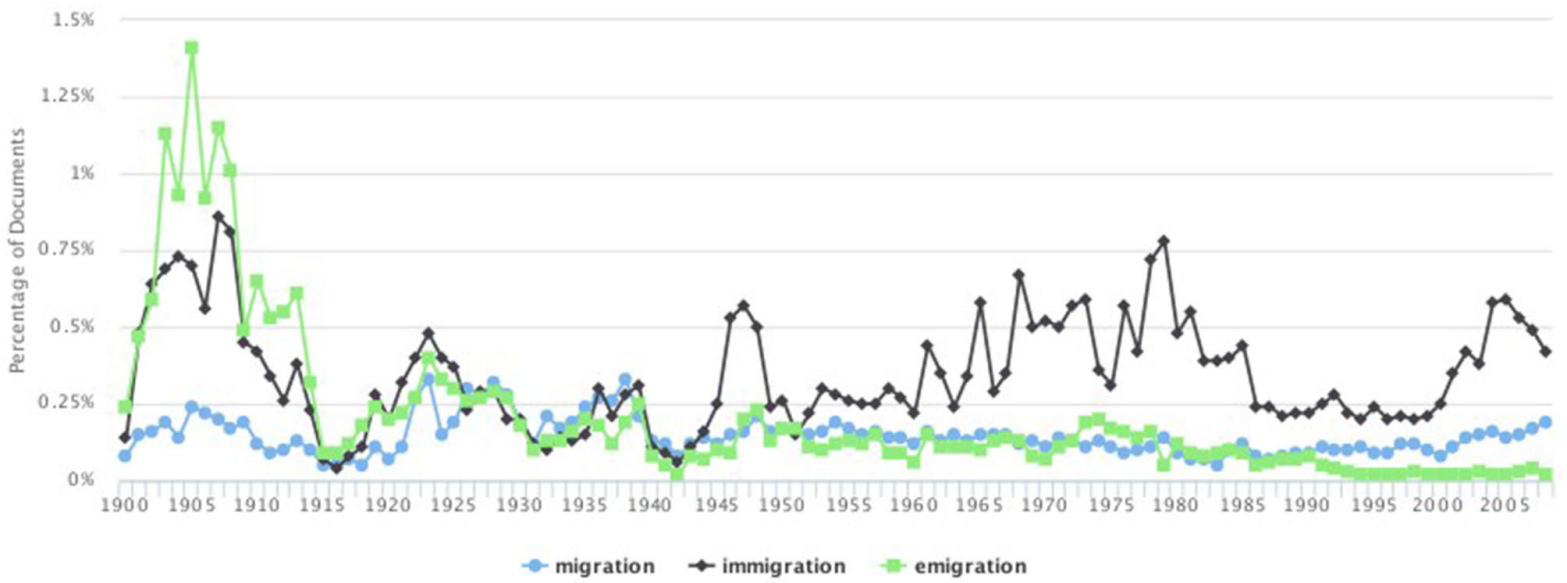
Percentage of total documents by year containing the terms *migration, immigration*, and *emigration*.

In this section, we demonstrate how the proposed method is applied by integrating DHA into the analysis of the word vector results. We have separated the discussion into five time periods following the quantitative results shown in Figure 1: 1900–1910, 1920–1930, 1945–1955, 1955–1985, and 1985–2000. The aim is not to explain historical changes or to confront historical knowledge with the computationally generated models, but to assess the value of including discourse-historical information into a computational model toward a greater understanding of the construction of meaning.

### Preliminary Data Exploration

The data were first explored by graphing the frequency of occurrence of the seed terms *migration, immigration*, and *emigration* in the timeframe of reference. This initial step was performed to obtain insights of patterns and continuities within the British discourse around migration in the long century. Figure 1 shows the percentage of the total documents by year that contains the terms of reference.

The graph yields interesting results. The first observation to be made concerns the fact that at the beginning of the century the three terms are sharply separated with a predominance of articles discussing *emigration*. This may indicate (1) that the migration discourse was not a generic topic incorporating the different aspects of human movement and (2) that emigration from Britain was discussed more often than immigration to the country and far more than migration in general. The second observation concerns the fact that between 1915 and 1940 this trend changes and the three topics seem to merge into one discourse. It is from 1945 that the current discourse polarization toward *immigration* can be first observed, whereas *migration* and *emigration* continue to converge until 1990, when they diverge again and *emigration* significantly decreases. This visualization of the frequency of the articles in *The Times* discussing the three topics already provides useful insights into how the wider migration discourse has developed in Britain: emigration, which used to dominate the discourse at the beginning of the century, has become, at the end of the observed period, the least frequently discussed topic. At the same time, in more recent times, the discourse has shifted almost completely toward immigration showing a clear change in the media construction of the migration discourse.

The graph is useful also to identify five main time slots or “word epochs,” which are marked by the spikes of the three terms' frequencies. The first spike is that of *emigration* and *immigration* at the beginning of the century (1905–1910); the second spike can be noticed between 1920 and 1930 when the three terms are used almost equally. It is in 1945, just after World War II (WWII), that the graph shows a sharp rise in *immigration* (third spike); between 1955 and 1985, although with some fluctuations, *immigration* keeps prevailing over the other terms (fourth spike), and finally, between 1985 and 2000s, the graph shows a significant decrease of the term *emigration*.

This initial exploration of the dataset offers useful starting points, which will be investigated more in-depth in the next stage of the study. Thanks to the word vector analysis, we will be able to look more in detail into the semantic space of the three terms at the times when the spikes occur. At the same time, the DHA triangulation will clarify how such changes in the frequencies may be understood in relation to the concurrent sociohistorical events.

#### 1900–1910

**Figure 2:**
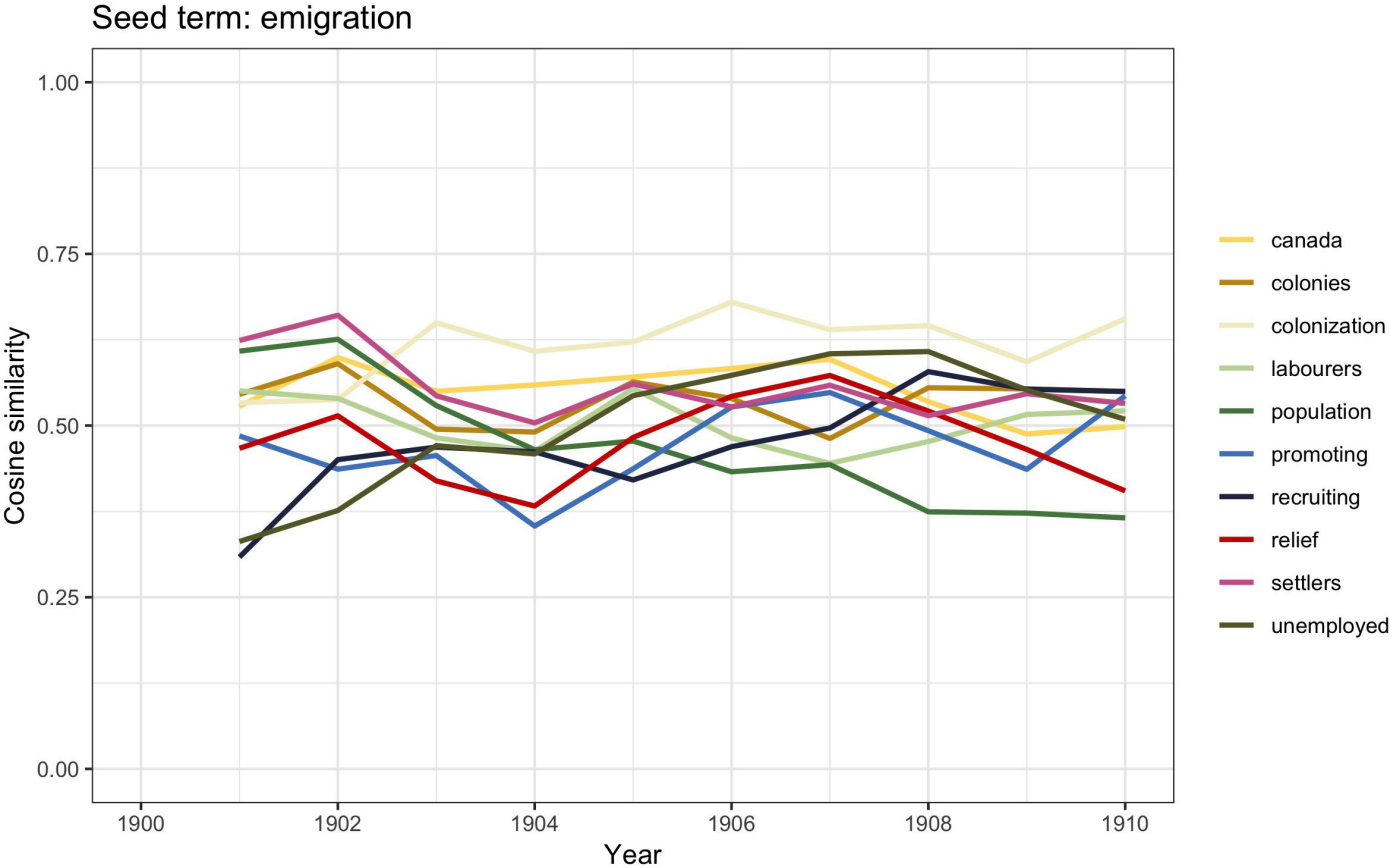
Similarity scores for the 10 most frequent word vectors in TDA for *emigration*, 1900–1910.

During the 1900s and early 1910s, British emigration was at its highest: it is calculated that in England and Wales as many as 8.7 per thousand and, in Scotland, 18.7 per thousand emigrated (Bueltmann et al., 2012), placing Britain among the European countries with the highest emigration rates^5^. It is therefore not surprising that in those years emigration had become a topic discussed in political and public debates. The graph in Figure 1 has already shown a peak in the number of documents containing the word *emigration*; Figure 2 visualizes the similarity scores for the 10 most closely related terms to *emigration* from 1900 to 1910.

Population growth and industrialization were the main factors for emigration. Industries such as the mining and textile had been severely affected by industrialization, and many workers had to face sudden unemployment; emigration was often seen as an obvious solution. The word vector similarities show words such as *population* and *unemployed*, which may suggest that emigration was often framed in the press as the easy answer to overpopulation and unemployment. It has already been noticed in the literature how the discussion would also sometimes incorporate imperial arguments according to which emigration was an efficient way to strengthen Britain's underpopulated colonies (Bueltmann et al., 2012). This would explain the presence of terms such as *colonization, colonies, settlers, recruiting*, and *promoting*.

The word vector similarities also show the word *Canada*. A DHA triangulation provides a potential explanation for this finding: between 1896 and 1914, Canada experienced rapid economic growth and development, thus becoming an attractive immigration destination. Estimates calculate that around 3 million immigrants arrived to Canada in those years, of which approximately one-third arrived from Britain (Lloyd, 2012, p. 137). Among other ethnic groups, British migrants were favored for several reasons: it was believed, for instance, that British immigrants would integrate more easily in Canada, as many Canadians already identified themselves as British. It was also believed that if British citizens had not moved to Canada in significant numbers, then Canada would be populated by “inferior” immigrants (Lloyd, 2012).

**Figure 3:**
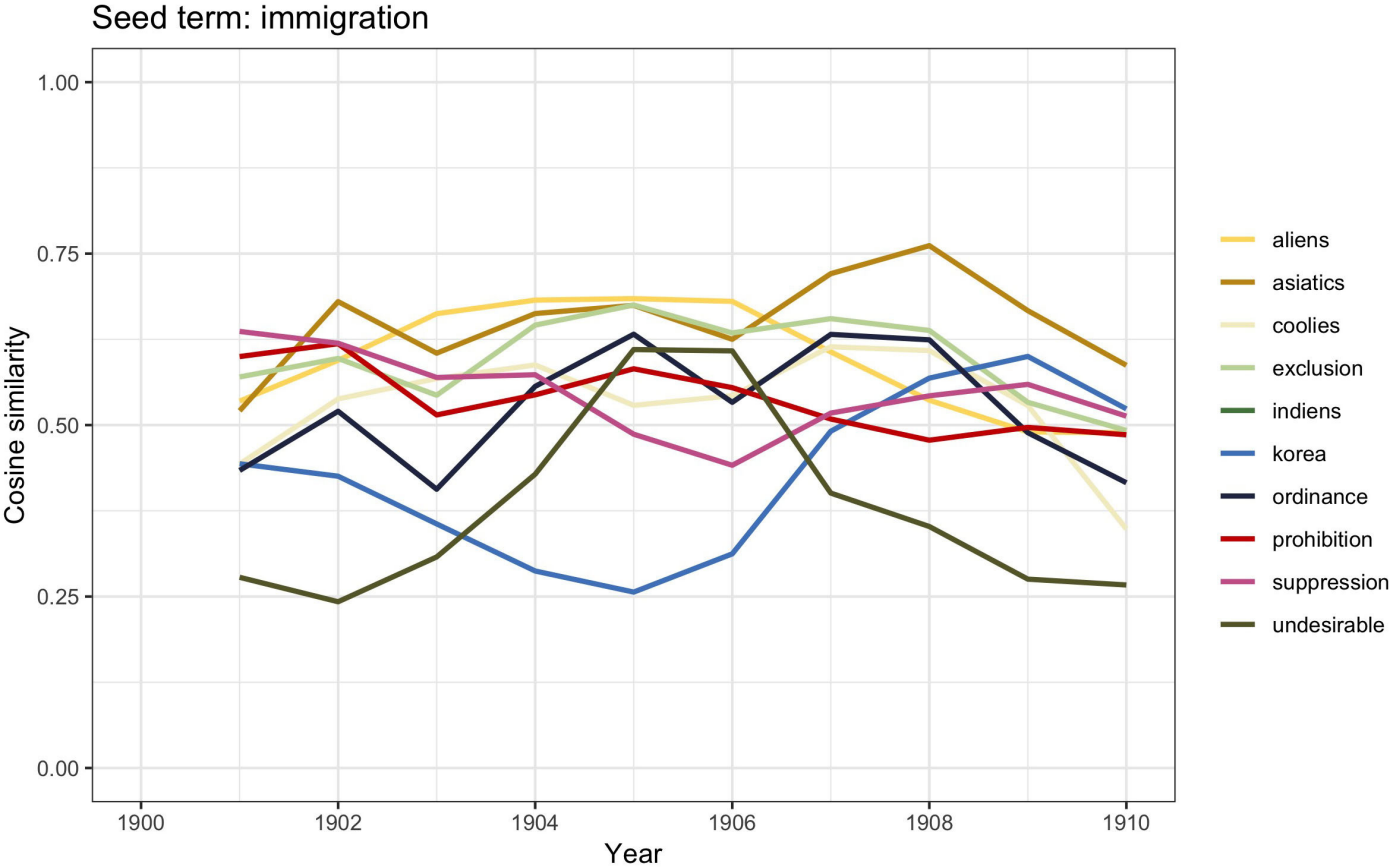
Similarity scores for the 10 most frequent word vectors in TDA for *immigration*, 1900–1910.

The graph in Figure 1 also shows a relatively high number of documents containing the word *immigration* between 1900 and 1910. If Britain's industrial boom was a cause for emigration, in the century before, it had also attracted hundreds of thousands of immigrants. Figure 3 visualizes the word vector similarity scores for *immigration*.

The analysis yields interesting results. The presence of words such as *asiatics, korea*, and *indiens* is not surprising: the opening of the Suez Canal in 1869 had facilitated immigration from India and China. However, the finding acquires a specific connotation if we look at the other terms: *exclusion, suppression, undesirable*, and *coolies*. By the end of the nineteenth century, anti-immigrant feelings were on the rise, and calls for immigration control laws became more and more pressing; the presence of terms with a stronger *relationship measure* (RM), i.e., the weight such as *prohibition, ordinance*, and *suppression* suggests such tensions. The xenophobic sentiments are apparent not only in the terms *undesirable* and *exclusion* but also in *aliens*, which most likely refers to the Aliens Act, entered into force in 1905. Finally, another important observation to be made concerns the absence of reference to the three largest groups of immigrants who had arrived to Britain in those years from Germany, Ireland, and southern Italy. This absence may suggest that at this point in history the hostility was mainly directed toward Asian immigrants.

#### 1920–1930

**Figure 4:**
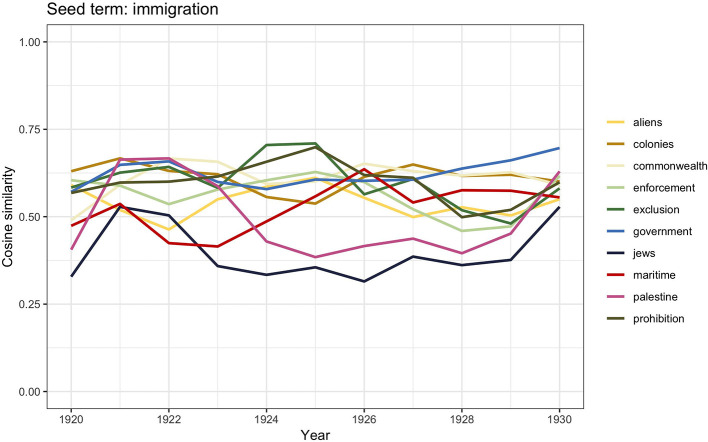
Similarity scores for the 10 most frequent word vectors in TDA for *immigration*, 1920–1930.

**Figure 5:**
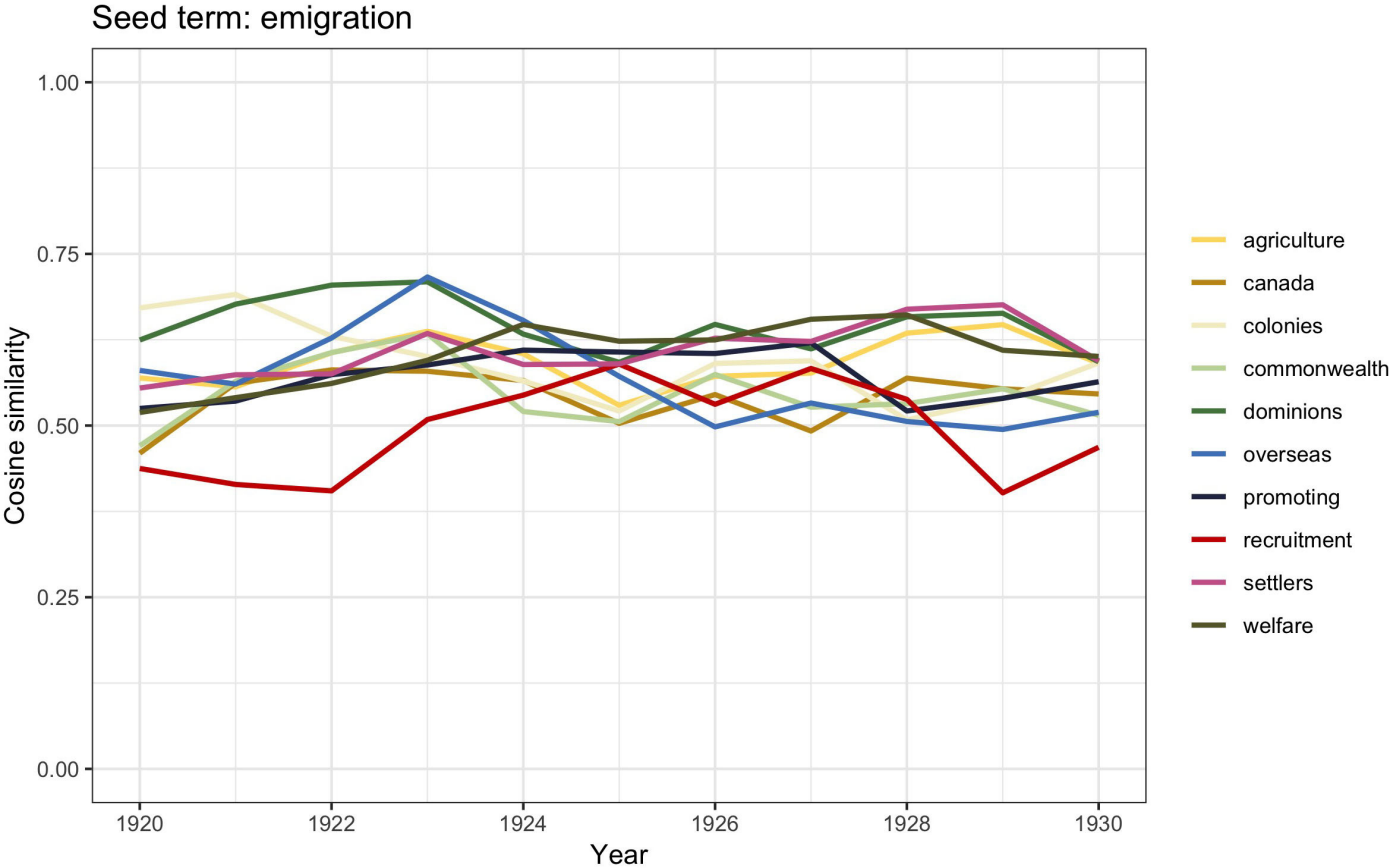
Similarity scores for the 10 most frequent word vectors in TDA for *emigration*, 1920–1930.

**Figure 6:**
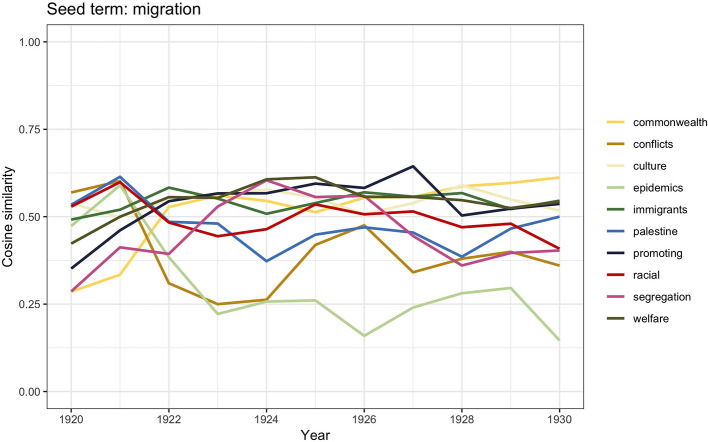
Similarity scores for the 10 most frequent word vectors in TDA for *migration*, 1920–1930.

The graph in Figure 1 shows how in the period between the end of WWI and 1930 the discourse incorporated all the three terms almost equally; we therefore computed word2vec similarities for all the three keywords. Figure 4 shows the results for *immigration*, Figure 5 displays the scores for *emigration*, whereas similarities for *migration* are visualized in Figure 6.

In those years, Britain was going through many social changes. Tension had arisen between white merchant seamen returning from war and migrant seamen, who in the meantime had replaced them. Violent riots and confrontations between the two opponents led to the proclamation of the 1925 Act which, by factually banning migrant seamen, became “the first instance of state-sanctioned race discrimination inside Britain to come to widespread notice” (Tabili, 1994, p. 56). In the time slot 1920–1930, the word vector similarities for *immigration* contain the words *maritime, exclusion, enforcement*, and *prohibition*. Meanwhile anti-Semitism was on the rise not only in Britain but also across Europe. Between 1882 and 1919, Jewish numbers in Britain had significantly increased from 46,000 to 250,000^6^; they were mostly escaping from Russia, where they were harshly discriminated. This may be related to the words *jews* and *Palestine* appearing in the similarities scores.

Emigration from Britain during the nineteenth and early twentieth century was primarily overseas; migration overseas was a major feature of Victorian society (Pooley and Turnbull, 1998, p. 258). It has been calculated that between 1840s and 1930s, people who emigrated overseas from Britain outnumbered those who migrated to Britain. The preferred emigration destinations were by far North America and Canada, but toward the beginning of the twentieth century, New Zealand, Australia, and South Africa had become more and more popular. This explains the presence of closely related words such as *Canada, colonies, commonwealth, overseas*, and *dominions*. The other words with high similarity scores are *promoting, recruiting*, and *agriculture*. Because of the greater distance and lesser knowledge of Australia and New Zealand, emigration to these countries was typically arranged by companies providing assisted passages. These companies would often recruit people whose sets of skills could benefit specific economic needs in the countries of destinations. This ultimately meant that these companies had control over the characteristics of those who moved (Richards, 1993; Haines, 1994; Pooley and Turnbull, 1998).

Although, traditionally, the majority of studies on race, ethnicity, and racism in Britain trace the beginning of xenophobic sentiments since 1945, some authors (e.g., Solomos, 2003) have stated that, in fact, it was during the interwar period that the question of racial difference started to enter the political debate of immigration. The social decay, particularly of seaport towns, started to be associated with black communities; shipping industry trade unions capitalized on this discriminatory belief and campaigned in order to restrict employment to white seamen. As we have already said, these discriminatory actions led to the 1925 Act; however, additional practices were reinforced, such as legalized different rates of pay based on race (Hepple, 1983, p. 44–45; Joshua et al., 1983), which were meant to prevent British citizens of a different race from settling in the country. But there was also another concern related to their settlement, and it had to do with the fear of a “mixed race” population as a result of mixed race unions (Rich, 1986, p. 120–44; Ramdin, 1999, 2017). The word vector similarity scores for *migration* reported in Figure 5 report the words *racial, conflicts, epidemics, immigrants, culture*, and *segregation*, which can be understood in the light of this historical contextualization. Because of the conflicts that would occur in some of the port towns, as well as the spread of an image of black people as sources of social problems, these communities were labeled as “aliens” and perceived as threatening to British culture (Solomos, 2003, p. 47). This set up the foundational arguments that characterized the political debate on “colored immigrants” following WWII.

#### 1945–1955

**Figure 7:**
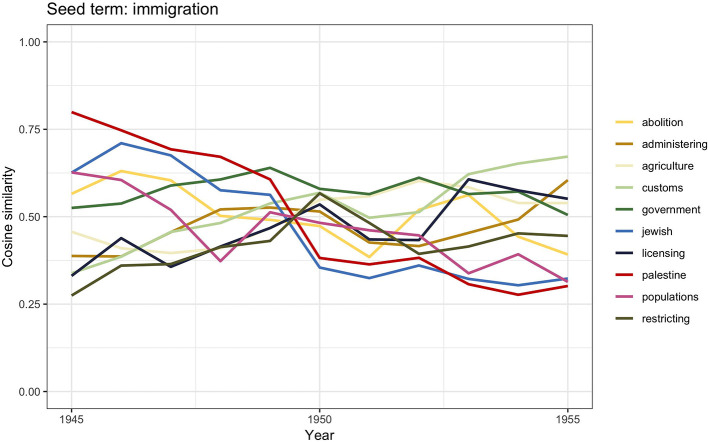
Similarity scores for the 10 most frequent word vectors in TDA for *immigration*, 1945–1955.

We will now move to analyze the third period between 1945 and 1955. The graph in Figure 1 shows that after WWII there was a peak in the number of documents discussing *immigration*, whereas *migration* and *emigration* showed a similar decreasing trend. As the similarity scores for *emigration* are very similar to those analyzed in the previous period, to avoid repetition in this section we comment the results for immigration (Figure 7). The full computed similarity scores are provided in the Supplementary Material.

The migration debate changed quite dramatically after WWII as more than 11 million people became displaced from their home countries throughout Europe, including former prisoners of war, released forced laborers, and survivors of concentration camps. The United Kingdom experienced considerable immigration from such displaced persons and other refugees from Europe. After 1945, the topic of immigration became connected with the persecution of the European Jews as the urgency of creating a new homeland for Jews in Palestine, debated throughout the war, had become a matter of public debate. This is visible in the word vectors similarity scores for *immigration*: *palestine* and *jewish*, which reflects the illegal immigration of Jews into Mandatory Palestine, governed by the United Kingdom between 1920 and 1948 (Kochavi, 1998; El-Eini, 2006; Cohen, 2014). It is interesting to notice that the term *palestine* significantly decreases after 1950 when the creation of Israel ended the British governmental involvement with Palestine. It is also worthwhile mentioning that the largest group to enter the United Kingdom between 1945 and 1954—the almost 1,000,000 Irish migrants—is not visible in the results. This would be in line with claims made by historians such as Solomos (2003, p. 42) who have highlighted how Irish immigrants hardly left a trace in public debate. Neither do we see references to Polish immigration, even though the secondary literature tells us that about 150,000 Polish army veterans were resettled in the United Kingdom between 1946 and 1949 (Sword, 1986; Blaszczyk, 2017).

The other terms appearing in Figure 7—*government, licensing, administering, restricting, abolitions*—may be understood in the context of the racialization of the political debate of immigration after the 1945s. Those are the years when the British government tried in many ways to prevent black migrants from entering Britain (Joshua et al., 1983; Carter et al., 1987), even when they were British. For instance, in 1948, the British Nationality Act distinguished between British subjects who were citizens of the United Kingdom and its colonies and those who were Commonwealth citizens, even though the right to enter and live in Britain was granted to both categories (Evans, 1983, p. 59–61; Evans, 1986). A number of nontransparent measures were additionally adopted to impede black immigration as much as possible (Carter et al., 1987). These actions mirror the sharp contrast between the government's intention of restricting the settlement of black colonial British citizens on the one hand and the wish to not undermine the notion of Britishness on the other (Joshua et al., 1983). According to Solomos (2003, p. 54) it was during this time that the political and media debate of immigration revolved heavily around race and color and on the effects that black immigration would have on the “racial character of the British people,” the *customs*, the national identity, and “Britishness.”

#### 1955–1985

**Figure 8:**
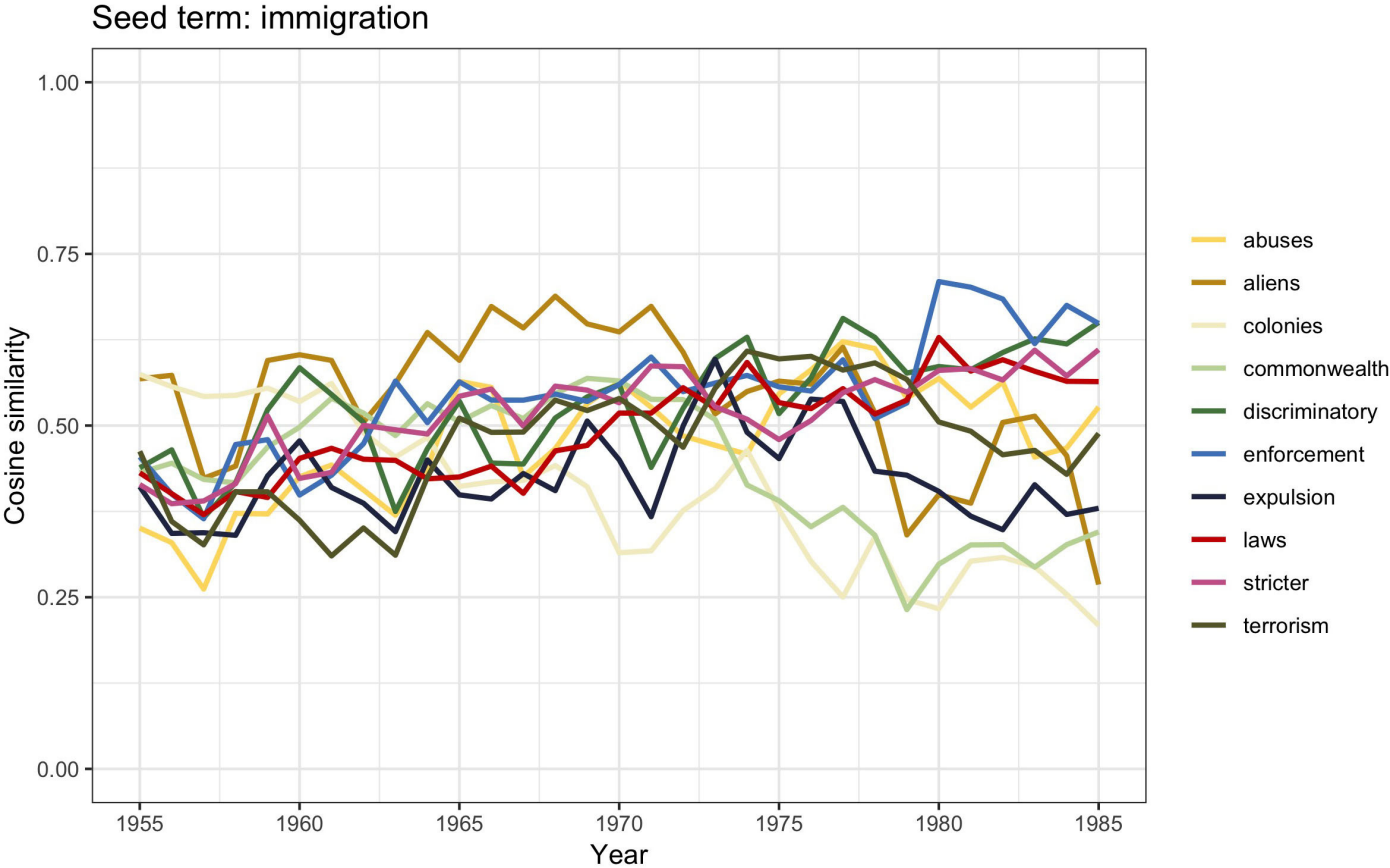
Similarity scores for the 10 most frequent word vectors in TDA for *immigration*, 1955–1985.

Throughout the 1950s, the British political and public debate of migration sharply polarized toward immigration, which was felt as a much more pressing issue than emigration. The graph in Figure 1 shows a clear peak for articles discussing *immigration* between 1955 and 1985; *emigration* and *migration* on the contrary displayed a similar low frequency. We here again discuss the similarity scores for *immigration* as shown in Figure 8.

The immigration debate of those years focused on the need to control “colored” immigration and revolved around two main arguments both aiming to legitimize the need to limit the number of nonwhite immigrants in the country. One argument concerned the urgent need for regulating immigration and called for active governmental interventions; this argument may be visible in the words *stricter, laws, enforcement*, and *aliens*. The other—visible in the words *colonies, commonwealth*, and *terrorism*—concerned the alleged social urgency of crime, employment, and housing in relation to too many colored immigrants in the country (Solomos, 2003, p. 53). At the same time, however, arguments against the introduction of more rigid controls were also raised both by conservative and labor politicians. Although it is not entirely clear which motivations were brought into the discussions, it seems that at least to an extent these measures were accused to be mere discriminatory practices (i.e., *discriminatory, abuses*) and cause for embarrassment to Britain as head of the Commonwealth and colonies (i.e., *Commonwealth, colonies*).

Eventually, all these arguments led to the 1962 Commonwealth Immigrants Act, which may be seen as the government's attempt to implement a measure that appeared to control immigration in general, whereas the real intention was in fact to limit black immigration only. The direct consequence of this Act was a high politicization of the term “immigration” itself, which *de facto* became code for racism (Solomos, 2003, p. 56). The 1962 Commonwealth Immigrants Act had in this way set the terms for the beginning of a political process enforcing even stricter immigration measures, such as the 1971 Immigration Act. With this Act, the notion of *citizenship* distinguished between partial and non-partial citizens, the former being the only ones having the right to reside in Britain. Factually, this measure was institutionalizing racism as it allowed only white Commonwealth citizens to enter and settle in Britain. From 1979, the Thatcher administration further strengthened the controls on immigration from the Commonwealth, starting from passing the 1981 British Nationality Act. This Act was dividing British citizenship into three categories: British citizens, British Dependent Territories Citizens, and British Overseas Citizens. The last category affected most British citizens of Asian origin who became in this way deprived of their right to live in Britain. This and other measures were justified by the argument that stricter immigration regulations were necessary to limit the number of people having access to social resources and services. However, by doing so, issues such as employment, housing, education, and law and order became highly racialized (Hall et al., 2013). Consequently, the focus of attention in the British public and political debate about immigration shifted from the question of immigration *per se* toward the identification of race as the source of the problem (Castles et al., 1984; Macdonald and Toal, 2014).

#### 1985–2000

**Figure 9:**
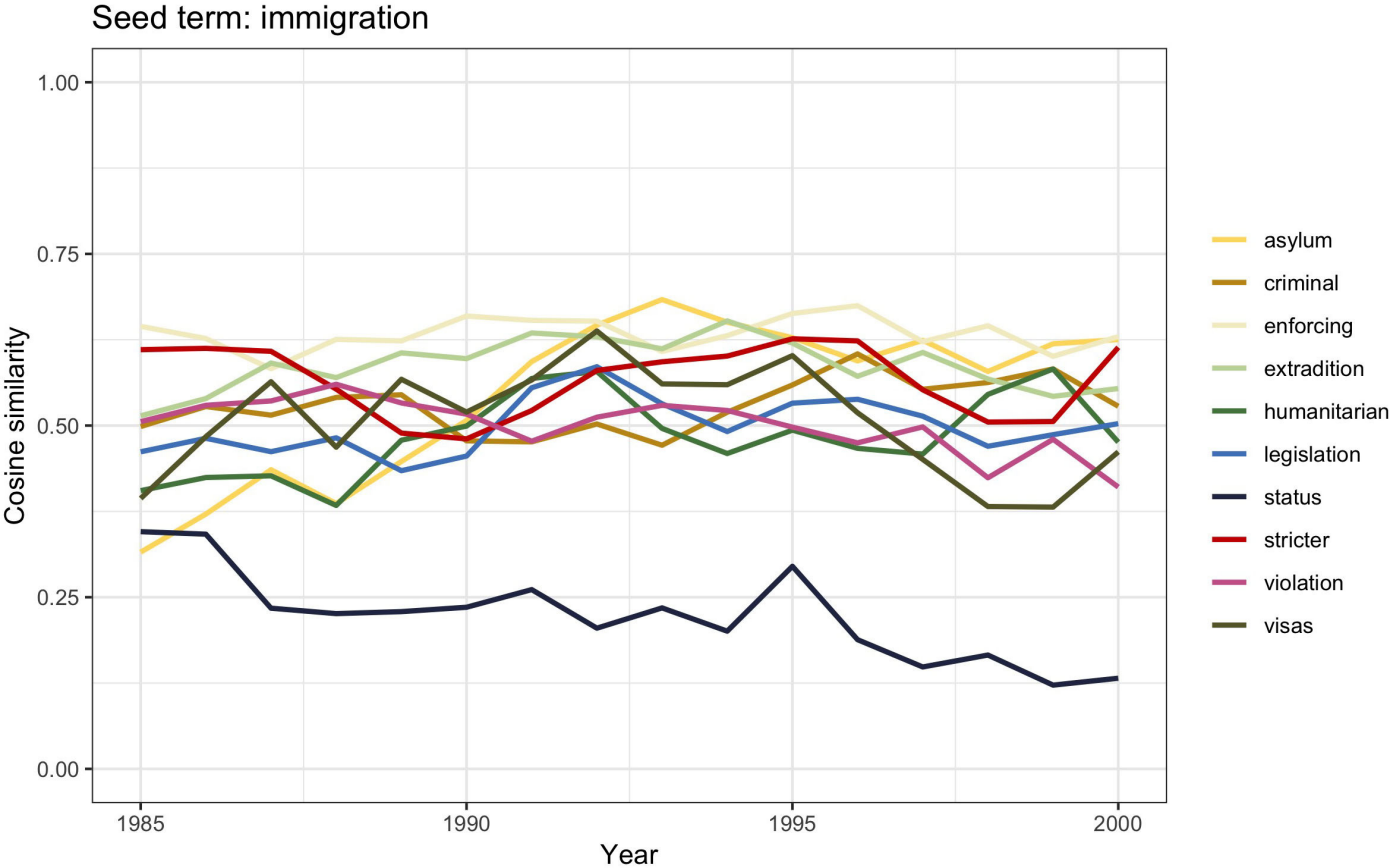
Similarity scores for the 10 most frequent word vectors in TDA for *immigration*, 1985–2000.

This section focuses on the last part of the century; the identification of the general debate of migration with immigration that had started in 1945 continued to the end of the century. The graph in Figure 1 reports an overall lower frequency of the number of articles discussing *migration, immigration*, and *emigration*, but it is once again *immigration* that shows the highest frequency. Figure 9 shows the similarity scores for *immigration*.

Partly due to the dismemberment of the communist bloc in Eastern Europe, the public debate during the early 1990s was dominated by discussions on asylum seekers and refugees. Such discussions soon became closely intertwined with the political and public debate on immigration—visible in the words *asylum, visas*, and *status*. Similarly to the arguments used only a few years before to legitimize stricter immigration measures against non-white immigrants, the focus of the debate was once again on the “alarming” growing number of asylum seekers and refugees in the country (Spencer, 1994, 1997; Macdonald and Toal, 2014). Such growing number was seen as a major concern for which stricter regulations were urgently required (i.e., *stricter, legislation, enforcing*). The discussions eventually led to the enforcement of the 1993 Asylum and Immigration Appeals Act, which aimed to reduce the number of asylum seekers and refugees able to claim sanctuary.

The presence of the words *violation, extradition*, and *humanitarian* could be explained by the events that led to the 1999 Immigration and Asylum Act. In 1996, the European Court of Human Rights had intervened on a specific deportation trial ruling that it was a case of human rights violation. The court's decision was effectively limiting the governments' power of deporting subjects for matters of national security. The British government's reaction was to set up a special commission that would work around the court's decision, essentially preserving the government's right to deport. The possibility to increase deportations together with harsher measures toward ethnic minorities and asylum seekers eventually constituted the core of the 1999 Immigration and Asylum Act.

In the last part of the century, the semantic connection between asylum seekers and refugees and the old concerns regarding the social and cultural dangers of immigration became tighter and tighter, reflecting the institutional legitimization of racial discrimination.

## Discussion

Unlike traditional distant reading approaches, the word vector similarity scores computed for 1900–2000 revealed trends and shifts that allowed for a wider contextualization and a much broader view than it would have been possible with smaller samples of data or analyses carried out over shorter periods of time. This demonstrates how computational distant reading reveals the *longue durée* of big history (Armitage and Guldi, 2014; van Eijnatten et al., 2014). Specifically, two macrotrends could be identified regarding the way immigration and emigration were discussed in the British public discourse. On the one hand, words such as *promoting, recruiting*, and *relief* were found associated with emigration *from* Britain suggesting that it was framed as a positive phenomenon, described as good not only for Britain but also for the emigration destinations of British citizens. The discourse-historical triangulation confirmed that emigration as the easy solution to overpopulation and unemployment, as well as an effective way to strengthen Britain's underpopulated colonies, was the main argument used to support this positive narrative. On the other hand, the opposite, yet consistent trend was found to be true for immigration *to* Britain, particularly from the colonies, which was consistently found in the context of negative terms such as *exclusion, undesired*, and *restricting*. Similarly to the way emigration was positively constructed through a range of legitimizing arguments, the construction of immigration as “negative for the country” was constructed through a variety of recurrent, yet powerful images: at times, it was associated with other social problems in the country (e.g., housing, unemployment), and at other times with the decay of British culture (e.g., loose customs) and with law and order issues (e.g., rise in crime, terrorism) or other general threats (e.g., invasion of immigrants, alarming numbers).

Another clear pattern was found within the semantic space of immigration and in the way in which, over the course of the century, immigration concerns became more and more associated with social categories of ethnic minorities (e.g., non-white seamen, Jews, immigrants from former colonies, asylum seekers), whereas larger groups of immigrants (e.g., Italians, Germans, Polish, Irish) were practically absent in the similarity scores. This could be an indicator of a process of racialization of the public debate around immigration, which, through a number of repeated arguments and narratives, targeted different categories of individuals at different times. The fact that immigration was embedded within widely debated issues of social urgency that required stricter laws and immediate intervention is also visible in the many terms referring to rules and regulations that were consistently present in the semantic space of *immigration* but that were totally missing in the similarity scores of *emigration*. This was found to be the case also when emigration from Britain had reached impressive figures, in fact, even when, historically, Britain was experiencing higher emigration than immigration. As emigration was seen as beneficial to the country, calls for more draconian measures referred exclusively to the immigration debate.

Word vector models are an extreme form of distant reading as the text structure itself disappears entirely; however, by integrating the technique with the discourse-historical triangulation, emerging larger patterns could be identified and understood. Although not optimal, the combination of very large quantities of data, the researcher's exclusion from the results, and the historical triangulation allows for a more empirical, reproducible, and comprehensive analysis that overcomes the limitations of either fully interpretative methods or conclusions drawn on fragmented data.

## Conclusions

This article offered a methodological contribution to the field of computational sociolinguistics. We combined neural word embeddings and methods of CDA to study the historical construction of public meaning at the level of discourse. The study's foundational hypothesis was that, because meanings are not established in isolation but are socially constructed, the analysis should move from word level to discourse level. To add historical depth and a broader contextualization, we also argued for a computational diachronic approach. In order to do so, we used word vector models built according to sliding time windows of one decade each and analyzed public discourse about migration in twentieth century Britain.

Our contribution was innovative in at least three aspects. First, by choosing to focus on migration as a *topic* rather than as a word—as it is typical in word embedding studies—we operated within a linguistic framework of conceptual history. This meant that while word vector models allowed us to trace the different vocabularies used in specific discourses over time, the discourse-historical angle provided us with the necessary framework to understand the correlation between language, meaning, and society. Second, the diachronic analysis allowed us to make sense of the variations and continuities in the discourse. Specifically, the examination of the observed changes within the historical context showed a recursive cycle: historical events were reflected in the public discourse; this, in turn, shaped explicit meanings in the public debate, which contributed to trigger further historical events. Third, the study focused on a specific type of discourse, *media discourse*. Mass media both reflect and influence public discourse as they are the main vehicle of knowledge-circulation and opinion-formation. Because they influence the way topics and events are perceived, mass media also impact both the public and policy makers. Thus, understanding media coverage of specific topics is essential not only to understand the corresponding society's response, but also to comprehend political and public attitudes that shape behavior, policy, and, finally, language. In reflecting collective discourse, media represent the common, shared knowledge that makes communication possible.

The study of public discourse about migration in the United Kingdom from 1900 to 2000 deepened our understanding of how meaning is constructed in language over time and how it shapes and it is shaped by sociohistorical change. The analysis revealed significant shifts in both the frequency and essence of how the meanings attached to *migration, immigration*, and *emigration* were formulated and discursively constructed as resulting from changing historical concerns in British society. For instance, at the beginning of the century, emigration was frequently discussed in the public media and promoted as an obvious solution to overpopulation and unemployment. A few years later, it was framed as an imperial necessity, the only way to strengthen Britain's underpopulated and “wrongly” populated colonies and dominions. After the devastation of WWII, migration was associated with displaced persons and the search for a Jewish homeland, and from the 1950s on, race and immigration became the dominant context of the great “migration debate.” Toward the end of the century, the dominant context of immigration as a publicly debated topic shifted again in the direction of internal social security and fear. These subtle shifts in the meanings attached to the migration discourse could be seen in the changing semantic space of the vectors and understood through the discourse-historical triangulation.

This research was based on the TDA. Although one of the most significant newspapers in the United Kingdom, *The (London) Times* is believed to have reflected English establishment, government, metropolitan interests, and empire. A fuller representation of public discourse would need to include other voices and, in the second half of the century, different media. Such a multimedia approach would offer a promising way to add a more comprehensive perspective to this line of inquiry. Nevertheless, despite its ideological, commercial, and political agendas, the collection remains an invaluable source for the study of public discourse. After all, in order to survive, newspapers must ultimately reflect contemporary debates of societal relevance.

Finally, the neural word embeddings used here should not be seen as a substitute for close reading strategies, rather as a complementary methodological approach that may allow researchers to adopt a zoom-out perspective to deal with large textual collections spanning over a long period of time. This macroperspective in combination with the discourse-historical triangulation may be used as a way to merge close and distant reading and proved effective at providing a comprehensive vision of the way meaning is socially constructed. By integrating these techniques, we also aimed to avoid confining the computational analysis to a role of support to critical analysis and to contribute to bridge the binary division between distant and close reading. This mixed method allows us to examine how language that is used to articulate public discourse is shaped by social changes and in turn may have helped to accelerate those changes. The study rested on the foundation that discourse conveys historical meanings. Therefore, understanding discourse changes means understanding social changes, and conversely, social changes will be reflected in changes in discourse. Although correlation does not prove direct causation, it is hoped that the method will highlight the importance of including sociohistorical data into a computational analysis so as to assist researchers to refine the quantitative results, make sense of them, and open avenues for understanding linguistic change.

## Data Availability Statement

All datasets generated for this study are included in the article/Supplementary Material.

## Author Contributions

This article is the product of a collaboration between historical linguist LV and cultural historian JV. All authors performed the historical and computational analysis and contributed to manuscript revision, read, and approved the submitted version.

## Conflict of Interest

The authors declare that the research was conducted in the absence of any commercial or financial relationships that could be construed as a potential conflict of interest.

## Footnotes

^2^A recent attempt to overcome such limitation has been proposed by *deep contextualized word embeddings* such as ELMo (Embeddings from Language Models; Peters et al., 2018), which go beyond traditional embedding techniques. The innovation consists in considering an entire sentence—as opposed to single words—before assigning an embedding to each constituting word. While this approach certainly marks an important step forward in NLP and appears to improve the performance of automated processes such as word-sense disambiguation, sentiment analysis, etc., it may not be the best approach to map the changing meaning of words over time as the problem of historicizing the meaning of the words in context remains.

^3^As a full review would have been beyond the scope of this article, we refer the reader to two recent excellent surveys carried out by Kutuzov et al. (2018) and Tahmasebi et al. (2018).

^4^For a recent account of the relationship between migration and media, see, for instance (Viola and Musolff, 2019).

^5^http://www.european-emigration.com/uk/

^6^https://www.bod.org.uk/jewish-facts-info/jews-in-britain-timeline/
